# Supplementary material for: Cloud BioLinux: pre-configured and on-demand bioinformatics computing for the genomics community
Source: BMC Bioinformatics. 2012 Mar 19;13:42. doi: 10.1186/1471-2105-13-42 (PMC3372431; doi:10.1186/1471-2105-13-42)
Supplement: Additional file 1 — Supplementary 1 Cloud BioLinux software documentation in the form of a mini, self-contained website. Users need to download and uncompress the .zip file, and open through a web browser the "index.html" file available on the main directory. (ZIP 1823 kb). [file 1471-2105-13-42-S1.ZIP › Cloud-BioLinux-Package-Documentation/docs/cap3.html]

Bio-Linux Software Documentation Pages

Back to search form

## cap3

|  |  |
| --- | --- |
| Name | cap3 |
| Description | **Cap3** is an assembly program. It is used to assemble sequences into contigs. On Bio-Linux, it is required by the ESTFerret program.  The Bio-Linux package contains only the cap3 binary. |
| Homepage |  |
| Remote Documentation | http://www.genome.org/cgi/content/full/9/9/868 |
